# The Association between Gut Microbiota and Osteoarthritis: Does the Disease Begin in the Gut?

**DOI:** 10.3390/ijms23031494

**Published:** 2022-01-27

**Authors:** Luciano C. Ramires, Gabriel Silva Santos, Rafaela Pereira Ramires, Lucas Furtado da Fonseca, Madhan Jeyaraman, Sathish Muthu, Anna Vitória Lana, Gabriel Azzini, Curtis Scott Smith, José Fábio Lana

**Affiliations:** 1 Department of Orthopaedics and Sports Medicine, Mãe de Deus Hospital, Porto Alegre 90110-270, RS, Brazil; lucianoramires@gmail.com; 2 Department of Orthopaedics, The Bone and Cartilage Institute, Indaiatuba 13334-170, SP, Brazil; drgabriel.azzini@gmail.com (G.A.); josefabiolana@gmail.com (J.F.L.); 3 Department of Biology, Cellular, Molecular and Biomedical Science, Boise State University, 1910 W University Drive, Boise, ID 83725, USA; rafaelapramires@gmail.com; 4 Department of Orthopaedics, The Federal University of São Paulo, São Paulo 04024-002, SP, Brazil; 5 Department of Orthopaedics, Faculty of Medicine, Sri Lalithambigai Medical College and Hospital, Dr MGR Educational and Research Institute, Chennai 600095, Tamil Nadu, India; madhanjeyaraman@gmail.com; 6 Department of Orthopaedics, Government Medical College and Hospital, Dindigul 624304, Tamil Nadu, India; drsathishmuthu@gmail.com; 7 Department of Medicine, Max Planck University Center, Indaiatuba 13343-060, SP, Brazil; annavitorialana1104@gmail.com; 8 Department of Medicine, University of Washington School of Medicine, Seattle, WA 83703, USA; css.smith53@gmail.com

**Keywords:** osteoarthritis, gut microbiota, metabolic syndrome, systemic inflammation

## Abstract

Some say that all diseases begin in the gut. Interestingly, this concept is actually quite old, since it is attributed to the Ancient Greek physician Hippocrates, who proposed the hypothesis nearly 2500 years ago. The continuous breakthroughs in modern medicine have transformed our classic understanding of the gastrointestinal tract (GIT) and human health. Although the gut microbiota (GMB) has proven to be a core component of human health under standard metabolic conditions, there is now also a strong link connecting the composition and function of the GMB to the development of numerous diseases, especially the ones of musculoskeletal nature. The symbiotic microbes that reside in the gastrointestinal tract are very sensitive to biochemical stimuli and may respond in many different ways depending on the nature of these biological signals. Certain variables such as nutrition and physical modulation can either enhance or disrupt the equilibrium between the various species of gut microbes. In fact, fat-rich diets can cause dysbiosis, which decreases the number of protective bacteria and compromises the integrity of the epithelial barrier in the GIT. Overgrowth of pathogenic microbes then release higher quantities of toxic metabolites into the circulatory system, especially the pro-inflammatory cytokines detected in osteoarthritis (OA), thereby promoting inflammation and the initiation of many disease processes throughout the body. Although many studies link OA with GMB perturbations, further research is still needed.

## 1. Introduction

Osteoarthritis (OA) has long been considered a degenerative disease that affects the hyaline cartilage alone. This orthopedic disorder still remains one of the most common degenerative and progressive joint diseases and a major cause of pain and disability in adults, affecting approximately 7% of the global population [1]. The Global Burden of Disease (GBD) 2019 study results revealed that the number of individuals affected by this condition increased globally by 48% between 1990 and 2019, classifying OA as the 15th highest cause of years lived with disability [2]. This significant increase is due to extrinsic factors such as the aging of the population as well as the indulgence in poor dietary habits [3,4,5]. Over the years, however, researchers started to notice that these pathological alterations were not exclusive to the chondral compartment. Adjacent structures including the subchondral bone, ligaments, synovium, and the joint capsule as a whole, are all involved in this pathological process albeit in varying degrees [6,7]. Many explanations have been proposed in attempts to fully elucidate the development of osteoarthritic alterations. Recent evidence indicates that OA progression is not exclusively attributed to biomechanical trauma but biochemical stressors as well, which may negatively affect regular activity of various cells and tissues [3]. Further research has shown that metabolic syndrome (MS), in particular, may in fact be one of the main culprits responsible for the development of OA. MS is a major health condition of modern-day society, and it only continues to expand and challenge public and clinical health on a global scale as a result of urbanization, increased calorie intake, the rise of obesity and sedentary life habits [8]. MS is connected to multiple physiological systems, being directly associated with the presence of four main clusters, which are: insulin resistance, obesity, vascular pathology, and dyslipidemia. MS paves the way for the progression of “meta-inflammation”, a state of persistent, low grade systemic inflammation triggered by metabolic stress [3]. This inflammatory stress disrupts cellular equilibrium and eventually aggravates systemic inflammation throughout the body [3]. By definition, meta-inflammation is a state of chronic inflammation mediated by macrophages present in multiple locations such as the liver, muscle, visceral fat, pancreas, colon and even the brain, for instance [9]. It is important to note that the state of chronic systemic inflammation also acts as a key mediator which drives the pathogenicity of OA by promoting harmful subchondral bone alterations in the onset of OA [10]. As a result, cartilage is also greatly affected by these alterations, aggravating inflammation even further, contributing to a shift towards a predominant pro-inflammatory and catabolic microenvironment in the joint [3]. Cytokines related to OA pathogenesis include tumor necrosis factor (TNF)-α, matrix metalloproteinases (MMPs), interleukin (IL)-1, IL-6, IL-2, IL-7, IL-15 and IL-21, and other chemokines which contribute to catabolic activity and detrimental effects [11,12]. These findings prove to be of particular significance since OA itself is influenced by the complex interplay between local, systemic and external factors, which consequently dictate disease progression and the manner in which patients respond to the treatment [13]. Despite its clinical and financial ramifications, conventional OA treatments still prove to be challenging. Conservative methods such as the administration of pharmacological agents only promote temporary alleviation of pain but do not address the etiological source of the disease and may, in some cases, cause serious adverse effects [7,14]. Pharmaceuticals may compromise the integrity of the gastrointestinal barrier, creating a state of hyper-permeability and inflammation [15]. As a matter of fact, long term administration of corticosteroids, for example, can increase the risk of serious side effects such as peptic ulcer disease, acute renal failure, and even myocardial infarction [16]. 

Recently, the gut microbiota (GMB) has caught the attention of medical experts for valid reasons. Although sometimes overlooked, time after time the GMB has presented itself as an essential component of human health and development, representing the totality of the microbial ecosystems that exist within and on the human body, including both organisms and their secreted products [17,18]. Research pinpoints a significant regulatory role of the GMB on neuroendocrine and immunological functions, thus demonstrating participation in many disease processes [19]. Recent investigations have provided data reinforcing a causal role for the GMB in bone homeostasis and thus OA initiation and progression. Many studies demonstrated murine models of joint degenerative disease mainly by establishing high-fat diet-induced obesity, mechanical over-loading, surgical induction, and even gene-editing techniques to make offspring susceptible to OA [20]. In all instances the GMB appeared to contribute to OA progression. In the context of human biology, studies have suggested that OA patients exhibit significant GMB dysbiosis, revealing a pathogenic OA-associated microbial shift [20,21,22]. This reinforces the concept that under biological stress the microbes in the GIT (gastrointestinal tract) can be pushed out of equilibrium, promoting pathological alterations that culminate in the manifestation of various disorders, especially OA.

The full involvement of the GMB in inflammatory and degenerative musculoskeletal disorders is being continuously investigated yet no consensus regarding mechanisms has been established. Deeper comprehension of how the multiple physiological systems interact and overlap may provide mechanistic insight into the development of OA and unfold feasible disease-modifying strategies. The question remains: could osteoarthritis really begin in the gut? In this review we aim to illustrate a possible link.

## 2. The Microbes Within: Good versus Evil

### 2.1. The Good

The human gastrointestinal (GI) tract caters more than 100 trillion micro-organisms [23] whereas the estimate on the density of bacterial cells in the colon is 10^11^ to 10^12^ per milliliter [24]. Under normal physiologic conditions, the gut microbiota exerts many vital functions in human hosts when it comes to the metabolism of nutrients and drugs, maintenance of integrity of the gastrointestinal mucosal barrier, immunomodulatory roles and even protection against exogenous pathogens [25]. Clinicians and researchers have learned that a healthy and stabilized gut flora becomes largely responsible for regular metabolic functions and thus the overall health condition of human hosts. As a matter of fact, increasing interest indicates that the scientific community is beginning to view the GMB as a sort of “secretory and modulatory” organ. A large scale study previously estimated that the human GMB contains approximately 35,000 bacterial species [26], but the two major constituent phyla predominating a healthy GMB are Bacteroidetes (*Bacteroides* and *Prevotella*) and Firmicutes (*Lactobacillus*, *Bacillus*, *Clostridium*, *Enterococcus*, *Staphylococcus*, *Ruminicoccus*, *Faecalibacterium*, *Roseburia*, *Dialister*, and *Sphingobacterium*) which constitutes 90% of the gut microbiome, followed by Actinobacteria (*Corynebacterium*, *Bifidobacterium*, and *Atopobium*), Proteobacteria (*Escherichia*, *Shigella*, *Desulfovibrio*, *Bilophila*, and *Helicobacter*), Fusobacteria (*Fusobacterium*), and Verrucomicrobia (*Akkermansia*) [24,27], which are known to be essential in maintaining a healthy microbe–host relationship [25]. Despite this major genetic profile, distribution of bacterial species can vary a lot from the beginning of the esophagus all the way down to the distal ends of the GIT. Remarkably, the large intestines contain over 70% of all the microbes found in the human body [25]. The human colon also provides a suitable home for primary pathogenic species such as *Salmonella enterica*, *Campylobacter jejuni*, *Escherichia coli*, *Vibrio cholera* and *Bacteroides fragilis*, yet in low proportions [28]. Currently, it is already well-established that age of the human (fetus, neonate, infant, child, adolescent, adult, or geriatric), GI tract (caecum, colon, rectum), gestational age (preterm or full term birth), type of delivery (natural labor or cesarean section), method of feeding (breast milk, artificial milk, supplementary feeds, or complimentary feeds), dietary habits, and antibiotic administration (clarithromycin, vancomycin, ciprofloxacin, or clindamycin) are notable variables which can transform and affect the GMB environment as a whole [27]. These transformations, in turn, can be either beneficial or quite detrimental depending on additional intrinsic and extrinsic factors that play out in distinct patterns on a per-patient basis [25]. 

For obvious reasons, the innate immune system is programmed to attack several micro-organisms which are not recognized as “self” components of the host. However, the truth is that most of the bacterial populations in the GMB are non-pathogenic. In fact, it is usually quite the opposite; they can co-exist harmoniously with other cells in the host in symbiotic fashion [25]. The symbiotic relationship of the GMB in the intestines, for instance, is much appreciated not only for the assistance of GMB in nutrient and drug metabolism, but also for their ability to act on the immune system in order to induce protective responses to prevent colonization and invasion by extraneous pathogens via the production of antimicrobial signals and competitive inhibition for nutrient and adhesion sites [29]. 

Speaking of nutrients, these microbes obtain their nutrients from fermented dietary carbohydrates. Colonic species such as *Bacteroides*, *Roseburia*, *Bifidobacterium*, *Fecalibacterium*, and *Enterobacteria* can ferment indigestible oligosaccharides and produce short chain fatty acids (SCFA) including butyrate, propionate, and acetate, which are rich sources of energy for the host in addition to playing vital health roles [30]. Butyrate, for example, prevents the accumulation of toxic residual products such as D-lactate [31]. Bacteroides, once again, are of particular research interest due to their renowned expression of glycosyl transferases, glycoside hydrolases and polysaccharide lyases, which are remarkable enzymes involved in carbohydrate metabolism. It turns out that the metabolic pathway involving carbohydrate fermentation also culminates in the synthesis of oxalate; however, additional bacterial organisms such as *Oxalobacter formigenes*, *Lactobacillus species*, and *Bifidobacterium species* can process and degrade oxalate in the intestinal tract, reducing risk of urinary tract complications [32,33]. 

Moreover, in terms of metabolic syndrome, these microbes also play an essential role in lipid metabolism. *Bacteroides thetaiotaomicron*, for example, can enhance the efficiency of lipid hydrolysis by increasing the expression of certain co-enzymes that work with pancreatic lipase during lipid digestion [34]. Lipid digestion and energy metabolism is an essential component that must be adequately regulated in order to prevent problems such as dyslipidemia, which is a key risk factor of metabolic syndrome. Elaborating further, members of this particular genus can synthesize conjugated linoleic acid, which is known to promote antidiabetic, hypolipidemic, antiobesogenic, and antiatherogenic effects, therefore being an extremely valuable player in the fight against metabolic abnormalities as a whole [35]. *Bacteroides intestinalis*, *Bacteroides fragilis* and *E. coli*, have demonstrated the ability to deconjugate and dehydrate the primary bile acids, converting them into the secondary bile acids such as deoxycholic and lithocolic acids, assisting in the maintenance of a healthy GI tract [36]. The secondary effects generated by gut microbes also allow increases in serum concentrations of metabolites including pyruvic, citric, fumaric and malic acids, which reflect increased energy metabolism [37]. 

Protein metabolism in humans is also partially attributed to the GMB due to the secretion of microbial proteinases and peptidases that play a cooperative role with human proteinases [25]. An example worthy of mention is the amino acid transport that occurs on the bacterial cell wall, in symbiotic fashion. As the amino acids from the intestinal lumen are passed into the bacteria, gene products end up converting these molecules into small signaling molecules and even antimicrobial peptides [38]. 

Another important activity is the processing of polyphenols, which humans often obtain from a balanced diet enriched with a variety of plants, fruits, and their associated products. Polyphenolic compounds such as flavanols, flavones, isoflavones, tannins, lignans, chlorogenic acids and anthocyanidins are absorbed in the intestines [25]. In general, these molecules are kept in their precursor forms as glycosylated derivatives bound to sugars until they reach the GI tract, where they are activated after removal of glycosylated structures by the GMB [39]. Interestingly, the structural specificity of the polyphenol molecules and the variety and richness in the gut niche strongly dictates the level of the processing capacity in the host’s intestine [25]. Once activated by the GMB, the activated polyphenols get absorbed by the intestinal circulation and subsequently delivered to distant tissues and organ systems in the body, where they will be able to promote additional healthy roles, such as antimicrobial properties [39].

**Figure 1:**
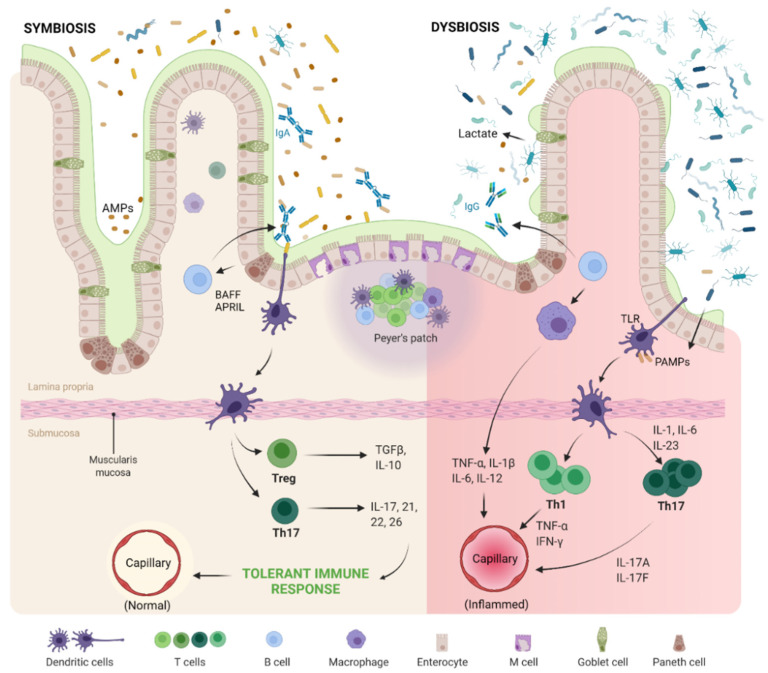
Mechanism of regulation of immune response by gut microbiome. The native immune system is tolerant to the resident gut microbiome under the tight control of intestinal epithelial cells using mucosal barrier, secretory IgA and antimicrobial peptides (AMP). The native gut microbiome stimulates the intestinal epithelial cells, dendritic cells and macrophages to activate the T regulatory (T reg) cells and T helper 17 (Th17) cells. Upon activation of the intestinal epithelial cells with toll-like receptors (TLRs), B-cell activating factor (BAFF) and a proliferation inducing ligand (APRIL) are secreted which promotes the differentiation of IgA producing plasma cells, whereas in dysbiotic status of gut microbiome with the loss of barrier integrity and breach in the intestinal epithelial cell barrier, translocation of bacterial components, pathogen-associated molecular patterns (PAMPs), intestinal immune system is triggered through TLR activation. This results in an inflammatory cascade through hyperactivation of T helper 1 (Th1) and Th17 cells resulting in section of inflammatory cytokines.

These gut microbe properties can produce significant protective effects in human hosts. The GMB has evolved considerably to the point where it is able to regulate and prevent overgrowth of specific pathogenic strains of bacteria. *Bacteroides*, once again, can activate dendritic cells, thus stimulating plasma cells in the intestinal mucosa to produce immunoglobulin A (IgA) in the host [40]. The IgA2 subclass, in particular, coats the GMB due to its increased resistance to degradation by the very own bacterial proteases that are released as a result of natural biological activity. This intelligent shift that leads to the formation of the protective IgA2 phenotype in the host is possible because the epithelial cells that line the intestines produce a cytokine called “APRIL” (a proliferation-inducing ligand) in (toll-like receptor) TLR-mediated bacterial sensing mechanisms [41]. This restricts the translocation of the microbiota from the intestinal lumen to the circulation, preventing the body from triggering a systemic immune response by inducing IgA producing plasma cells, as shown in Figure 1. These immunoglobulin producing plasma cells of the gut release TNF-α and iNOS for GMB stimulation which further induces the secretory IgA function of B cells [42]. Having these boundaries properly demarcated, the GMB can safely participate in the induction of synthesis of additional peptides such as cathelicidins C-type lectins, and defensins by Paneth cells via pattern recognition receptor (PRR)-mediated mechanisms, without causing any adverse immunological reactions [43]. Specific molecular patterns and their cross-talk, such as the PRR-MAMP (pattern recognition receptor-Microbe Associated Molecular Patterns), can lead to the activation of signaling cascades that promote mucosal barrier function and production of antimicrobial peptides (AMPs), mucin glycoproteins and the aforementioned IgA [44]. This proves to be essential for the proper maintenance of the GMB, being of great benefit for both the host and the gut microbiome themselves. Hence, GMB controls and maintains the symphony and homeostasis between microbial population and the host, as shown in Figure 1.

### 2.2. The Evil

Recently, under many different circumstances, dysbiosis of the GMB has appeared to be associated with a vast array of health conditions. These range from luminal disorders including inflammatory and irritable bowel diseases, metabolic syndrome (mainly obesity and insulin resistance), and hyperallergic reactions all the way to even nervous system problems, as it has been linked to neurodevelopmental illnesses as well [45,46,47,48]. As mentioned earlier, the predominant phyla that inhabit the large intestine are Bacteroidetes and Firmicutes. The Firmicutes:Bacteroidetes ratio has been implicated in predisposition to disease states such as obesity, metabolic syndrome, low grade inflammatory arthritis, inflammatory bowel syndrome, and neuropsychiatric diseases [24]; however, significant variability is found even in healthy individuals, making researchers reconsider this idea. Earlier, it was estimated that the number of bacterial cells present in human GMB far exceeds that of its host by approximately ten-fold [49], whereas Abbott reported that for an average human, the ratio for microbial cells to human cells is 1.3:1 [50]. Sender et al. recalculated the bacterial load to human cells as 1:1 which does not produce any significant difference in the microbiota [51]. There is currently enough evidence in the literature that illustrates a link between metabolic syndrome and dysbiosis-associated chronic low-grade inflammation [52]. The mechanism of GMB dysbiosis resulting in systemic inflammation is depicted in Figure 1. The most significant factors that can cause a shift in the GMB are diet and genetics, which can cause major alterations in intestinal integrity and promote metabolic endotoxemia. This ultimately renders the patient more susceptible to obesity, insulin resistance and glucose intolerance due to the activation of TLR4 (toll-like receptor) and subsequent production of inflammatory cytokines. The GMB-derived metabolites such as SCFAs and Gram-negative bacterial lipopolysaccharides (LPS) can exert anti-inflammatory and pro-inflammatory effects, respectively, by acting on macrophages which helps in tissue homeostasis. Though they are primarily associated with antimicrobial functions, they are quite sensible to biochemical signals and display polarization of their two main phenotypes. These phenotypes are the M1 subtype, which takes on a more pro-inflammatory role due to microbicidal activity, whereas the M2 subtype is associated with more anti-inflammatory and reparative roles [6]. The macrophage is strongly associated with GMB and therefore is also implicated in pathogenesis of various diseases, even more so the ones that are triggered by meta-inflammation, as shown in Figure 1 [3,9,53,54].

Obesity, for example, which is also a state of low-grade chronic inflammation, is characterized by expanded adipose tissue (AT) in which resident macrophages may account for almost 30% of the total composition [55]. The increased number of adipose tissue resident macrophages (ATMs) in AT is mainly attributed to the increased recruitment of blood-macrophages and augmented proliferation of macrophages that are already present. This is where the problem begins. ATMs are known to be major effectors of AT inflammation. These cells undergo a tendency to accumulate in this specific biological environment and ultimately trigger activation of inflammatory pathways, increasing the secretion of pro-inflammatory cytokines [56]. Linking back to the GMB, scientists learned a some time ago that the composition of the GMB from lean and obese mice show major differences. The dysbiosis caused by obesity in mice promotes a shift, generating a lower proportion of *Bacteroidetes* and higher *Firmicutes* bacterial populations [57], illustrating a link between a “sick” GMB and the development of obesity as well as low-grade chronic inflammation [52]. Further evidence indicates that alterations in the GMB are responsible for this inflammatory progression via two principal pathways. Metabolic syndrome (more specifically obesity) can cause a decrease in *Bifidobacterium* levels and lead to a reduced production of Glucagon-like peptide-2 (GLP-2), which is vital for the maintenance of intestinal barrier function. This means that low concentrations of this molecule compromise the tight junction integrity of the epithelial barrier in the GIT, increasing intestinal permeability and therefore provoking a condition often referred to as “leaky gut syndrome”, as some researchers choose to call it [58,59]. Consequentially, LPS then infiltrates the circulatory system and the complex blood vessel network through passive diffusion across the intestinal mucosa. This predicament has also been found to further contribute to macrophage accumulation in AT [60,61]. ATMs recognize gut-derived LPS via TLR4 receptor signaling on the cell surface, which in turn causes the conversion of the M2 to M1 phenotype and subsequently the increased secretion of major pro-inflammatory cytokines such as IL-1β and TNF-α [62]. Some authors also report that the lack of TLR4 receptors in experimental mice models attenuates AT inflammation due to the predominant macrophage shift toward the M2 phenotype [63]. Regarding the immunological perspective of the GIT, researchers previously identified the CD14+ macrophage, a CD-specific subset that expresses high levels of the CD14 protein and produces pro-inflammatory cytokines such as IL-23 and TNF-α, leading to the accumulation of inflammatory macrophages and mediators in the gut [64].

## 3. GBM-Derived Metabolites and Osteoarthritis (OA) Progression

Recent attention has been given to bacterial-derived LPS, specifically, as this microbial protein has been increasingly implicated in inflammatory disorders, namely OA. Researchers have revealed a correlation between elevated levels of circulatory inflammatory biomarkers (including LPS) with the severity of OA, therefore painting GMB-derived metabolites as pathogenic mediators responsible for driving inflammatory musculoskeletal disorders [65,66]. For instance, an animal study demonstrated that mice on a 28-week high-fat and high-sugar diet developed an obese phenotype and displayed increased cartilage damage, establishing a direct correlation between serum LPS levels and Mankin histological scores [65]. In this study the authors also examined GMB composition via 16S sequencing, detecting significant increases in *Lactobacillus* and *Methanobrevibacter* bacterial species, which indicated that MS promoted a strong dysbiotic shift in murine GMB with a strong predictive relationship with histological scores. In a similar study, Ulici et al. were able to demonstrate reduced severity of post-traumatic OA in germ-free mice, implying once again a causal role for the GMB in musculoskeletal pathogenesis [67]. Most of the animal studies evaluating the impact of GMB were performed on rodents due to the similarity of their GMB to that of the human gut microenvironment [68,69]. 

A similar pathogenic process occurs in humans. Dysbiosis of gut microbiome promotes excess porosity in the epithelial barrier of the gut and leakage of microbes and their by-products into the circulation, as shown in Figure 1 [70,71]. Stress involved in metabolic syndrome and pain involved in OA modulate gut microbiota through release of neurotransmitters and result in increased intestinal permeability [71,72]. 

The hypothesis behind the dysbiosis of gut and the development of OA are (a) low-grade intestinal inflammation [73,74], (b) elevated levels of microbial lipopolysaccharide (LPS) [75], (c) metabolic endotoxemia (interaction of gut-derived LPS and toll-like receptor (TLR)-4) [76,77], (d) meta-inflammation (metabolic inflammation mediated by macrophages present in multiple locations such as the liver, muscle, visceral fat, pancreas, colon and even the brain) [73,78,79], and (e) metabolic syndrome (abdominal obesity, dyslipidemia, hypertension, insulin resistance ± glucose intolerance, pro-inflammatory and prothrombotic states) [80,81]. The presence of inflammatory products and microbial genetic products in the joint pose a temporal association between gut microbiota and arthritis [20,82]. 

### 3.1. Gut–Joint Axis Distortion

“Gut–Joint” axis establishes the crosstalk between gut and joint [20,83,84,85,86]. Gut microbiota elaborates the wide range of metabolites, enzymes, and short chain fatty acids. These microbes produce lipopolysaccharides (LPS) which pave a way for increased intestinal permeability (“leaky gut”) and enter into systemic circulation to produce chronic low-grade intestinal inflammation. With respect to LPS, there exists an association with obesity and metabolic syndrome that are the potential risk factors for the development of OA. There is proven evidence of the role of LPS in OA pathogenesis [87]. Dunn et al. [88] revealed the identification of microbial DNA signatures in articular cartilage of rodents and humans. The authors performed 16S ribosomal RNA gene deep sequencing on eroded and intact cartilage samples from knee and hip OA patients, analyzing microbial DNA diversity and metagenomic profiles. The findings in human cartilage were compared to those in cartilage from OA-susceptible and OA-resistant mice. Result analysis indicated that alterations in microbial DNA signatures occur during OA progression. Although knee samples were microbiologically distinct from hip cartilage, microbial DNA in OA individuals was associated with increased Gram-negative constituents. This evidence shows that gut dysbiosis leads to the progression of the natural course of OA [76].

The role of MS on gut–joint instability in the absence of obesity has recently been investigated. In a mouse model of MS, Guss et al. [89] analyzed the effects of mechanically-induced OA on TLR5-deficient mice. Much like previous findings, histological evidence indicated that severe changes in cartilage were present in the high-fat diet mice groups, corresponding to GMB dysbiosis, increased body fat and systemic inflammation (as expected), only this time with an increased number of Firmicutes bacteria. Although metabolic irregularities were found in TLR5-deficient mice, the authors concluded that, in isolation, they could not have been solely responsible for the development of OA. Actually, the increased levels of LPS and the overgrowth of Firmicutes played a much more expressive role, here revealing a strong correlation between microbial components and OA progression. 

### 3.2. Gut–Joint–Brain Axis Distortion

Turroni et al. established Gut–Joint–Brain (GJB) axis with OA pain [82]. The altered pain perception in OA cases is due to the modulation of the peripheral nociception and sensitization phenotype which results in the discrepancy in the results of the estimation of OA pain to the severity of radiological findings [90]. Increased intestinal permeability allows microbial metabolites to prime macrophages and exacerbate the joint inflammation, resulting in pain [91]. With the existing “Gut–Joint” axis, the exposure of stress and pain alter the interactions between the brain and the intestine, resulting in distorted quorum sensing signals and microbial gene expression, altered GI secretion, increased gut permeability and mobility, and dysbiotic gut. All this disequilibrium between gut microbiota and pain perception results in joint and systemic inflammation [92,93]. Understanding the temporal relationship among the pain perception in OA, gut microbiota, and joint inflammation leads to improved therapeutic strategies in the management of patient health in OA. 

### 3.3. Evidences on Pathogenesis of OA

In a study involving 25 patients with knee OA, researchers were able to establish a link between serum and synovial fluid levels of LPS with known hallmark features of OA: the presence of activated macrophages (M1—pro-inflammatory) in the knee joint capsule and synovium; joint space narrowing; osteophyte formation; and high WOMAC (The Western Ontario and McMaster Universities Arthritis Index) scores, indicative of severe pain [66]. This also lies in parallel with a larger cohort study in the Dutch population [94]. The Rotterdam study-III recruited 1444 patients with hip and/or knee OA, where a solid association between increased WOMAC scores and abundance of *Streptococcus* bacteria with pro-inflammatory profile was found. For these reasons, physicians have been prompted to view the GMB from a new perspective, as a patient’s GMB must also be accounted for in consideration of possible dysbiotic shifts and secondary pathogenic effects. 

Coulson et al. compared 3000 mg/day of green-lipped mussel (GLM) and 3000 mg/day of glucosamine (GS) in OA patients for 12 weeks and evaluated therapeutic efficacy on gut microbiota. In the GLM group, increased Bifidobacterium and decreased Enterococcus and yeasts were observed whereas in the GS group decreased Bacteroides and increased yeasts and coliforms, most notably Escherichia coli, were observed. Clostridia was reduced in both the groups, which is a potent immunomodulatory that decreases inflammation, improved WOMAC and GSRS scores and improved OA symptoms in response with colonic Th17 and CD4+ regulatory T cells [95].

Boer et al. evaluated gut microbiome and joint pain and inflammation. They demonstrated a spurious association between increased amounts of *Streptococcus* spp. and higher OA-related knee pain, but the causal association needs to be established. The possible hypothesis for OA-related knee pain and *Streptococcus* spp. is due to the production of microcellular vesicles by *Streptococcus* spp. in the GI tract. With the above findings, by reversing the gut dysbiosis through diet interventions, OA-related knee pain can be reversed. The causal association between *Streptococcus* spp. and OA has to be establish before translating into clinical practice [91].

Huang et al. demonstrated the role of LPS, a pro-inflammatory mediator from Gram-negative microbes, in accelerating the severity of OA [87]. They established a correlation between the presence of LPS in synovial fluid in the knee with the increased activated macrophages in the knee and clinical and radiographic severity of OA knee [17]. 

## 4. Interventional Strategies: Fixing Dysbiosis

**Figure 2:**
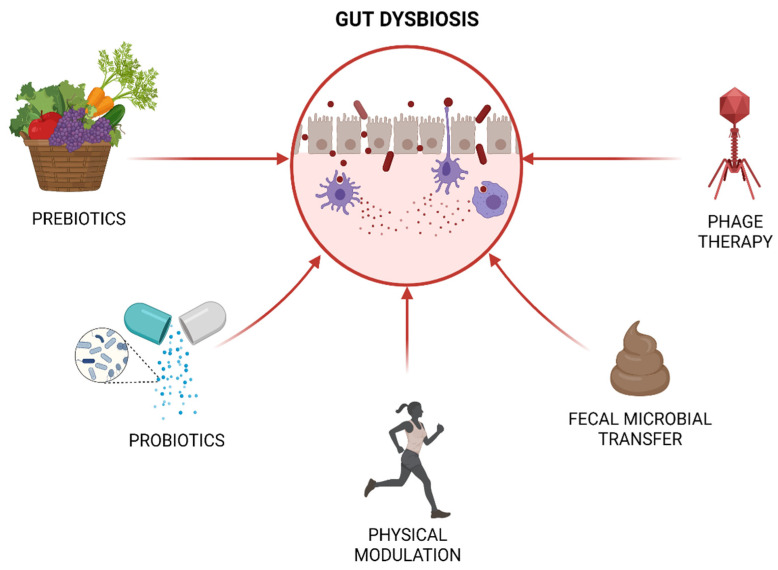
Interventional strategies to counteract dysbiotic gut microbiome.

In light of these facts, physicians have attempted to propose the regulation of “gut illness” via different methods such as nutritional supplementation, physical exercise programs and other alternative biological approaches, as shown in Figure 2 [96].

### 4.1. Prebiotics

Perhaps the cheapest and most viable alternative would be to modify the patient’s lifestyle by targeting their dietary habits and encouraging them to include more “GMB-friendly” foods on a daily basis. It is well-known that diet still plays a key role in shaping the composition, diversity, and richness of the GMB even in adulthood. A diet rich in a wide variety of fruits, vegetables and fibers is associated with a higher richness and diversity of gut microbes [25]. The ingestion of prebiotics and probiotics, for instance, is known to be very beneficial for gut health [97]. In 2017, the International Scientific Association for Probiotics and Prebiotics (ISAPP) defined prebiotics as a substrate that is selectively utilized by host micro-organisms conferring a health benefit [98]. Prebiotics are a group of selectively fermented ingredients that promote specific alterations in the composition and activity of gut bacteria, thus providing benefits to the host by improving overall health [97]. In order to be classified as prebiotic, a compound must be the following: resistant to the acidic pH of the stomach; unsusceptible to hydrolysis and absorption in the GIT; able to undergo fermentation by the gut microbes; and capable of selectively stimulating the growth and activity of the GMB with beneficial effects towards the host [97]. There are different types of prebiotics but the majority of them are part of carbohydrate groups, being mostly oligosaccharides. It should be noted, however, that prebiotics are not limited to carbohydrates only. A fitting example would be cocoa-derived flavanols, which, according to in vivo and in vitro studies, convey stimulatory effects on lactic acid bacteria [99]. As for the carbohydrate groups, the most common types include galacto-oligosaccharides, fructans, and other oligosaccharides derived from starch, pectin and glucose [100]. In overview, these compounds effectively stimulate the “good” bacteria and increase the production of gut metabolites, which shape the GMB and therefore provide the host with secondary beneficial effects [100]. More specifically, these dietary compounds can induce SCFA synthesis, affecting cell proliferation and differentiation, production of hormones, and inflammatory modulation, which can be very beneficial for osteoarthritic patients [15]. A more complete set of their roles is found in Table 1. Due to the “Western Diet” in the form of high calorie and fat intake, low fiber uptake leads to disorganization of gut microbial ecology which is linked with the development of various autoimmune and inflammatory diseases [101]. In an equilibrium, host and gut microbiota exist in mutualistic relationship [102]. When the disruption occurs in this mutualistic relationship, several inflammatory and autoimmune diseases manifest [103,104,105]. Intestinal epithelial cells (IECs) are responsible for immune-regulatory functions and possess a major influence on the growth and homeostasis of immune cells [106,107]. IL-10 produced by IEC preserve the gut epithelial intactness from epithelial-macrophage hit and protect the microbiome [108,109].

Gut microbiome and mucosal immunity interactions are influenced by dietary fiber and short chain fatty acids (SCFAs) [110]. The gut microbiome ferments non-digestible polysaccharides to yield SCFAs such as acetate, propionate, and butyrate [111]. The products of SCFAs possess metabolic homeostasis and anti-inflammatory effects to prime the immune system [112,113]. SCFAs works via G-protein-coupled receptors (GPCRs) or reduction of histone deacetylases (HDACs) and provide a protective environment to host and the GMB [112,114]. The component acetate, a metabolite of SCFA, produced by the GMB, decreases gut permeability which is a significant feature of probiotic bacteria and extends safety from other pathogens [110]. The concept of “leaky gut” in humans and rodents provides an increased gut permeability, microbial disequilibrium, and reduced mucosal immunity, which changes the mucosal barrier action and hence attributes to the development of inflammatory and autoimmune diseases [115,116].

GPR109A is a G-protein-coupled receptor for nicotinate but recognizes butyrate with low affinity [117]. The gut microbiome possesses anti-inflammatory effects through the GPR109A signaling pathway and induces colonic macrophages and dendritic cells to potentiate the differentiation of T reg cells and IL-10-producing T cells [118,119]. Due to low affinity of butyrate to be sensed by GPR109A, it is crucial for the colonic epithelium to maintain gut viability. Coupling with NF-κB pathway, GPR109A inhibits pro-inflammatory cytokines such as IL-1β and IL-6, iNOS, COX2, and TNF-α [120]. Hence, GPR109A acts as a therapeutic target to manage inflammation. Hence, dietary fiber re-organizes the gut microbiome to improve inflammasome and upregulates through their attachment to GPR109A [37,121].

### 4.2. Probiotics

Unlike prebiotics, probiotics are living organisms found in specific dietary supplements which may also ameliorate host health [15]. The FAO/WHO defined probiotics as the live micro-organisms which when administered in adequate amounts confer a health benefit on the host [122]. There are studies in the literature which describe the therapeutic potential of probiotics in the treatment of inflammatory disorders, especially the ones of arthritic nature. Probiotics appear to have control over inflammatory diseases depending on the bacterial strains and species [15]. To elaborate, So et al. [123] have shown that the oral administration of *Lactobacillus casei* conveys protective effects against rheumatoid arthritis in rodents. This micro-organism suppresses collagen-induced arthritis, reduces paw swelling, and attenuates lymphocyte infiltration and the destruction of cartilage [123]. Furthermore, the authors also report its ability to upregulate IL-10 whilst reducing the expression of pro-inflammatory cytokines IL-1β, IL-2, IL-6, IL-12, IL-17, interferon-gamma (IFN-δ), cyclooxygenase 2 (COX-2) and TNF-α. These effects are associated with suppression of exacerbated Th1 immune responses seen in arthritic inflammation. In similar fashion, Amdekar and colleagues [124] demonstrated the beneficial effects of a daily dose of 2 × 10^8^ CFU of *Lactobacillus casei* per milliliter of distilled water in a collagen-induced model of arthritis in rats. This strategy reportedly prevents synovial infiltration, pannus formation, as well as the destruction of bone and cartilage with a significant reduction in pro-inflammatory cytokines, thus being effective against the state of progressive inflammation. In a double blind, randomized placebo-controlled study [125] of rheumatoid arthritis, 30 patients were assigned in order to assess the efficacy of a probiotic formula containing *Lactobacillus reuteri* and *Lactobacillus rhamnosus* over 3 months. The probiotics were administered orally twice daily in encapsulated form, containing 2 billion CFU per capsule. Although the probiotics did not show a statistically significant improvement in disease activity, there was evidence indicating attenuated secretion of pro-inflammatory mediators including TNF-α, Il-1α, IL-6, and IL-15.

Similar benevolent effects are also seen in OA. In a rat model of OA, for instance, So et al. [126] further demonstrated that when orally co-administered with type II collagen and glucosamine six times per week, *Lactobacillus casei* can significantly reduce pain, cartilage destruction and lymphocyte infiltration in comparison to isolated treatments. *Lactobacillus casei* and glucosamine cooperatively decrease nuclear translocation of NF-κB in chondrocytes and decrease IL-1β, IL-2, IL-6, IL-12, IL-17, TNF-α and IFN-δ and MMPs 1, 3, and 13, whilst upregulating expression of ILs 4 and 10, which are anti-inflammatory.

Mechanisms of action for probiotics have previously been introduced in the literature. These bacteria appear to dictate a range of physiological functions in the GIT that influence immune responses, epithelial barrier function and cellular proliferation [127]. Some of the known effects include the following: antimicrobial action via the assembly of inhibitors of gene expression in pathogenic strains; competitive inhibition for binding sites against pathogens; inhibition of virulence gene or protein expression in GIT pathogens; and stimulation of immune responses due to the increase of sIgA and anti-inflammatory cytokines and the rescue and regulation of pro-inflammatory agents [127]. 

### 4.3. Physical Modulation

Physical activity is another factor that must be incorporated into a patient’s lifestyle in order to promote additional health benefits. Multiple animal studies have previously demonstrated that exercise training independently alters GMB composition and functionality [128,129,130,131,132,133,134,135,136]. The primary findings of Matsumoto et al. [135] revealed that voluntary running exercise alters microbiota composition and increases butyrate levels in mice. This is of vital importance to both the host and the GMB because butyrate serves as the primary fuel for colonocytes, allowing an increase in colonic epithelial cell proliferation, amelioration of gut barrier integrity, and regulation of host immune system and gene expression [137,138]. 

In humans there is also evidence illustrating the role of exercise in shaping the GMB. A study designed by Clarke et al. [139] revealed that the intestinal microbiota of elite rugby players exhibited greater alpha diversity and higher abundance of 40 different bacterial taxa in comparison to the microbiota of lean sedentary controls. Additionally, the sportsmen also had lower abundance of Bacteroides and Lactobacillus species. A recent similar study [140] in women found that physically active females who performed at least 3 h of exercise per week had greater levels of *Faecalibacterium prausnitzii*, *Roseburia hominis*, and *Akkermansia muciniphila* in comparison to sedentary controls. These findings are of significant value to physicians since *F. prausnitzii* and *R. hominis* are two bacterial species that produce butyrate whilst *A. muciniphila* is associated with lean body mass index and improved metabolic health [141,142]. It should be noted, however, that these are cross-sectional studies which could not be adjusted for the effects of diet, which is known to affect the GMB, as previously discussed. For instance, the dietary habits of physically active individuals can vary significantly in comparison to sedentary patients. In the study of Clarke et al. [139], specifically, professional rugby players consumed large amounts of protein and this factor can be accountable for the observed differences in the microbiota. This therefore means that, in some cases, such effects may not be exclusively linked to exercise alone, at least in the aforementioned scenarios. Perhaps a combination of a protein-rich diet with an adequate exercise program may promote synergistic effects which are of great benefit to both the GMB and the host.

Probable mechanisms regarding the role of exercise on GMB modulation have been proposed. It is known that the gut-associated lymphoid tissue (GALT) extends from the small to large intestine, containing approximately 70% of the immune cells present in the body [143]. Findings from previous animal studies revealed that exercise can alter the gene expression of intraepithelial lymphocytes, not only decreasing the production of pro-inflammatory cytokines but increasing the secretion of anti-inflammatory cytokines and antioxidant molecules as well [144,145,146]. To elaborate, the immune cells present in the gut are found in proximity to microbes and produce antimicrobial agents which play important roles in regulating host-microbial homeostasis. Additionally, exercise can affect the integrity of the mucus layer in the GIT. This not only prevents microbes from adhering to the gut epithelium but also serves as substrate for specific microbes such as *A. muciniphila* [143]. High-intensity physical activity can temporarily increase intestinal permeability and the contact between microbes and immune cells, which can be reduced at resting states or regular exercise intensities. In comparison to sedentary individuals, trained athletes generally have lower levels of circulating bacterial endotoxin LPS at rest and a greater heat-shock protein (HSP) response to heat stress due to intense physical activity [147,148]. Higher concentrations of HSP in the gut can promote beneficial effects as they have been shown to prevent the degradation of tight junction proteins between epithelial cells [149]. 

Interestingly, a recent animal study [150] led by Meng et al. further revealed that exercise under sustained cold conditions promotes a significant shift in the GMB composition of obese mice and enhances the browning of white adipose tissue and weight loss. These findings suggest that physical modulation, as a whole, acts by means of hormetic stress in the gut. Microbial populations as well as the host’s own immune system receive multiple stimuli, generating beneficial adaptations and amelioration of the integrity and resilience of the gut barrier and its physiological functions. The global changes in the GIT due to exercise may affect the intestinal pH, mucus secretion, biofilm formation and nutrient delivery to gut microbes [143]. 

Another feasible and perhaps novel strategy to stimulate the GMB is whole body vibration (WBV). WBV is a non-invasive physical therapy which is often included in patient rehabilitation programs [151]. This technique mimics mild effects of physical activity on the body and is capable of improving performance in athletes and even rehabilitating the musculoskeletal tissues of astronauts due to the prolonged absence of gravity [152]. 

Although not fully elucidated, WBV can also reduce inflammation and significantly improve metabolic health [153]. WBV machines work by providing rotational or vertical vibratory stimuli to the body. Vertical vibration generates an upward thrust, alternating with gravity. This produces rapid up and down forces, providing constant biomechanical stimuli to multiple organs and tissues [154,155]. In the GIT, more specifically, WBV has been shown to promote benevolent effects in the control of inflammation. A recent animal study led by Yu et al. [153] revealed that WBV induces the polarization of M1 to M2 in diabetic mice, which contributes to the maintenance of inflammatory homeostasis and attenuation of dysbiosis. Additionally, WBV is also responsible for alterations in the fecal microbiome, increasing Bacteroides, especially the ones belonging to the *Alistipes* genus of the Rikenellaceae family. The recent findings of Song et al. [151] revealed additional effects of WBV therapy on intestinal microbiota. In their study, mice and human volunteers were placed on a WBV platform for 30 min daily, over 30 days. Upon analyzing immunological results, the researchers found that WBV influences the differentiation of regulatory T (T reg) cells, a process which is usually reflective of changes in the GMB. Microbiome analysis revealed that this modality also affected the intestinal microbiota. as the content of *Lactobacillus animalis* was significantly elevated in response to vibration in mice. On the other hand, the contents of *Lactobacillus paraplantarum* and *Lactobacillus sanfranciscensis* in the human body suffered significant alterations. The WBV-mediated alterations in intestinal microbiota composition regarding *Lactobacillus* spp. were linked to the differentiation of T reg cells in mice and physical characteristics in humans. These cells play an essential role in the human intestine as they produce IL-10, which is vital for the maintenance of intestinal homeostasis and GMB equilibrium [151,156,157]. Lactobacilli are abundant micro-organisms in the human GIT and are known to be associated with good intestinal health [158]. Animal studies have demonstrated that Lactobacilli play various roles associated with the prevention and management of infectious diseases. As an example, it can attenuate interleukin-1β-mediated inflammation and improve barrier function, thus revealing its potential to halt and reverse intestinal damage during infection [159]. These findings suggest that this alternative non-invasive approach may be much more viable for patients who, for whatever reason, are unable to engage in regular physical exercise programs, especially those who are already suffering from musculoskeletal inflammation.

### 4.4. Fecal Microbiota Transplantation

Fecal microbiota transplantation (FMT) is an alternative therapeutic approach which consists in collecting fecal matter with stable gut microbes from a healthy donor and transplanting it into the GIT of a patient with a perturbed GMB in order to re-establish equilibrium in the microbial community [160]. In order to diminish risks, medical experts must first select a suitable donor with no history of significant disorders by screening the individual for metabolic syndrome, pathogenic microbes, autoimmune disorders or other malignant diseases. Once the initial screening is done, fecal matter is collected and prepared by mixing with water or normal saline, which is then filtered in order to remove potential debris [160]. Lastly, the filtrate can then be administered via esophagogastroduodenoscopy, retention enema, colonoscopy, or even through a nasogastric or nasojejunal tube [160]. This therapy emerged decades ago for the treatment of *Clostridium difficile* infections, but only recently was FMT able to gain more popularity due to its feasibility in the management of complicated gastrointestinal disorders [161,162]. 

Although further studies are still needed, FMT appears to be broadening its application well beyond local intestinal perturbations, serving as a potential therapeutic intervention for other complications such as metabolic syndrome, neuropsychiatric disorders, autoimmune conditions, allergic diseases, and even specific types of tumors [161,163]. Since gut dysbiosis is mostly attributed to the overgrowth of pathogenic microbes, the development of new antibiotics has proven to be highly challenging. Not only can this therapy kill off non-pathogenic species but also create additional threats with the emergence of antibiotic-resistant pathogens, which have a dense microbial population and increased opportunities for horizontal gene transfer [162]. The flora in feces has been regarded by some as a special organ, which led researchers to view FMT as a procedure similar to organ transplantation [164]. Nevertheless, FMT is now considered an important therapeutic modality in the manipulation of gut dysbiosis. Transplanted feces from healthy donors can deliver a richer and more stable community of intestinal micro-organisms as well as proteins, bile acids, and vitamins, which collectively contribute to the reharmonization of standard gut function [165]. The bacterial strains from the donor and the associated antimicrobial components produced by them, such as adhesins, can compete for sites with pathogenic strains in the recipient. This prevents the pathogenic microbes from colonizing the intestine and causing further damage, which is the core mechanism underpinning FMT therapy [166]. 

**Figure 3:**
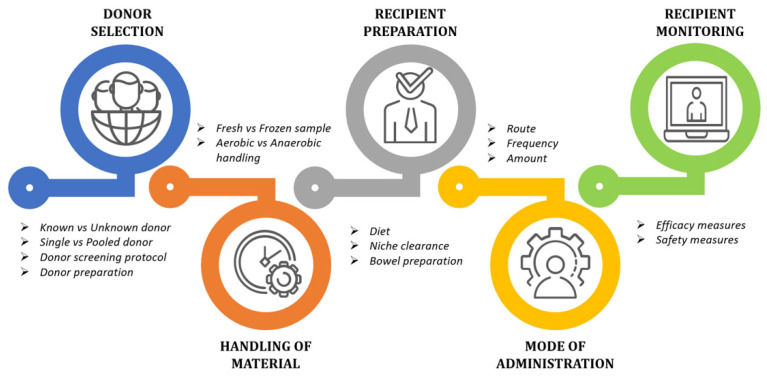
Challenges in Fecal Microbiota Transplantation (FMT) therapy.

In the case of OA, preventing the overgrowth of pathogenic strains and the release of toxic substances associated with disease progression is an imperative approach. However, there are various challenges that need to be resolved before successful implementation of a standardized FMT for OA knee cases, as shown in Figure 3.

### 4.5. Bacteriophage Therapy

In 1917, Felix D’Herelle coined the term bacteriophage, which was a hypothetical viral agent responsible for the decreased bacterial load. In the 1940s, the mechanism of phage-mediated killing was understood and recognized for treating infections. The usage of phage therapy persisted within Soviet Medicine in Georgia and was practiced routinely in the USSR [113]. Bacteriophage therapy is a more advanced therapeutic tool which employs the use of bacterial viruses to treat bacterial infections [167]. This modality is quite old and emerged long ago also as a response to the declining effectiveness of antibiotics. The approach relies on the use of phages, which are naturally-occurring viruses measuring approximately 25–200 nm, to infect and lyse bacteria [167]. With the advances in medical biotechnology, researchers have been able to enhance this process by creating bioengineered phages and their lytic proteins in order to target multidrug-resistant microbes, proposing the efficacy of this novel biological approach against the emergence of microbial threats [167]. The several advantages associated with the implementation of this strategy include low toxicity to humans, biofilm degradation, host-specificity and self-amplification [168]. These “nano-machines” are the most abundant biological entities on Earth, with an estimated number between 10^31^ and 10^32^ phages. They play an important role in regulating the ecosystem by keeping bacterial populations under control. As an example, they are responsible for the death of approximately 20–40% of marine surface bacteria every day [169]. In the past, much controversy was raised due to limited knowledge and documentation of these biological agents and their variable success in human health, which would explain why this therapy was only able to gain more significant reputation in the scientific community after the invention of electron microscopy [170]. Since phages are naturally-occurring bacterial parasites, they compulsorily rely on a bacterial host for survival. Their life cycle consists in binding to specific receptors on bacterial cell surfaces and introducing their genetic material into the cell. “Lysogenic” phages can integrate their genetic information into the host genome for vertical replication (mother to daughter cell), whereas “lytic” phages hijack the cellular machinery to produce the next generation of phage progeny and ultimately lyse the cell. 

**Figure 4:**
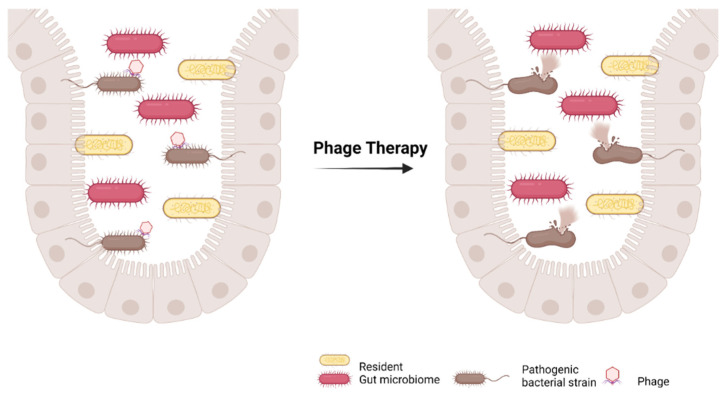
Traditional phage therapy for gut dysbiosis with pathogenic microbiome.

Additionally, in accordance with microenvironmental stimuli, lytic proteins can become activated and react with the peptidoglycan cell wall, causing hydrolysis and the subsequent release of novel phage particles to reinitiate the cycle [167,171]. It is worthy to note that most phages are infectious only to the bacterial cells that express the matching receptor. There is variation among phages in regard to host-specificity, as some of them are limited to specific strains whereas others have a more sophisticated arsenal, being able to overcome biological resistance and infect a range of bacterial strains and even genera [172,173]. Traditional phage therapy is strongly based on the application of lytic phages, which ultimately lyse and destroy the bacterial host as shown in Figure 4. In order to prepare a patient for treatment, lytic phages must be compiled into a mixture referred to as “phage cocktail”, which contains various phages with a demonstrated in vitro efficacy against the specific pathogen [167]. Due to the bacteriophage’s specificity for certain kinds of bacteria, phage therapy may demonstrate the therapeutic potential to manipulate and rearrange the dysbiotic GMB niche, restoring microflora balance [174]. More specifically, medical experts can screen the patient and accurately identify the pathogenic strains that are causing problems and recruit specific phages that can selectively attack the culprit [174]. Although promising, there are certain hurdles associated with this approach, such as the requirement of the selective induction of lysogenic phages, their ability to persist in the gut due to various factors, and the immune responses triggered in the host [175,176]. In order fit in with current health and safety guidelines, phage therapy must lack the ability to integrate its genome into the genome of the target bacteria or any of the components of the GMB niche in a way that causes interference with the standard microbiota functions [177]. Secondly, it must not affect the GMB through selective pressure on non-target microbes or the target of infectious agents. This leads to the development of resistant offspring which can subsequently disrupt the equilibrium in surrounding beneficial bacteria as well as the host’s evolution and adaptability mechanisms against phages themselves [177]. This field in medical biotechnology is still undergoing expansion and remains to be further explored as the literature proves to be often scarce and contradictory.

## 5. Conclusions

The gut microbiota plays crucial roles in homoeostasis by regulating metabolic pathways, nutrient metabolism and, consequently, the synthesis of many other products that are implicated in additional biological activity, especially immunomodulatory functions. The microbial species that colonize the gut can be either protective or pathogenic depending on specific conditions provided by the host. Currently, there are many strategies that can fix GMB dysbiosis. However, the most obvious and perhaps cheapest strategy to revert detrimental GMB alterations would be to simply remove the inadequate dietary components and encourage the patient to engage in some form of physical activity. Common prebiotics and probiotics present in many healthy foods can significantly stimulate the proper function of symbiotic microbes which produce metabolites, vitamins and other proteins that are essential for human health. When proposing treatments for musculoskeletal disorders, especially OA, physicians should consider alternative angles and closely monitor the GMB of a patient. Instead of solely focusing on bone and cartilage, medical practitioners should be twice as vigilant about the patient’s metabolic status. Due to the complexity of human physiology, the root of a specific pathology may not always be so obvious. Although there is a significant quantity of studies establishing a solid correlation between the GMB and OA, more clinical investigations are needed in order to fully elucidate the complex interplay between intrinsic and extrinsic factors that affect the composition and behavior of the microbes within.

## Figures and Tables

**Table 1:** Common types of prebiotics and their functions.

Prebiotics	Functions
Fructans	Selectively stimulates lactic acid bacteria.
Galacto-Oligosaccharides	Stimulates *Bifidobacteria*, *Lactobacilli*, *Enterobacteria*, *Bacteroidetes*, and *Firmicutes*.
Starch and Glucose-Derived Oligosaccharides	Elevates butyrate production and stimulates *Bifidobacteria*.
Other Oligosaccharides (pectin-derived)	Strengthens the mucus layer, enhances epithelial integrity, and activates or inhibits immune cells.
Non-Starch Oligosaccharides (flavonoids)	Inhibits the growth of pathogens, increases the number of Bifidobacterium and Lactobacillus, reduces endotoxin production, converts bile acids, maintains gut homeostasis, and promotes nutrient absorption.

## Data Availability

Not applicable.
